# Associations between tobacco inhalation and semen parameters in men with primary and secondary infertility: a cross-sectional study

**DOI:** 10.3389/fendo.2024.1396793

**Published:** 2024-05-14

**Authors:** ShiWei Fan, Zeling Zhang, HuiRu Wang, Lei Luo, Bo Xu

**Affiliations:** Reproductive Medicine Center & Department of Obstetrics and Gynecology, The First Affiliated Hospital of University of Science and Technology of China (USTC), Division of Life Sciences and Medicine, University of Science and Technology of China, Hefei, Anhui, China

**Keywords:** primary infertility, progressive sperm motility, secondary infertility, semen quality, smoking, sperm concentration

## Abstract

**Objective:**

To examine the impact of tobacco smoking on seminal parameters in men with both primary and secondary infertility.

**Methods:**

This cross-sectional study analyzed 1938 infertile men from China who were categorized as nonsmokers (n=1,067) and smokers (n=871), with the latter group further divided into moderate smokers (1-10 cigarettes per day) (n=568) and heavy smokers (>10 cigarettes per day) (n=303). We assessed semen volume, concentration, total sperm count, progressive motility, and normal morphology following World Health Organization (WHO 2010) guidelines. A logistic regression model was used to analyze the relationships between smoking and seminal parameters while also controlling for lifestyle factors.

**Results:**

The analysis demonstrated a statistically significant correlation between smoking and adverse seminal parameters in both primary and secondary infertility patients. Specifically, primary infertile men who smoked had a lower semen concentration, with heavy smokers showing a median sperm concentration of 59.2×10^^^6/ml compared to 68.6×10^^^6/ml in nonsmokers (P=0.01). The secondary infertile men who smoked exhibited reduced forward sperm motility, with heavy smokers demonstrating a median progressive motility of 44.7%, which was significantly lower than the 48.1% observed in nonsmokers (P=0.04).

**Conclusion:**

Smoking is significantly associated with detrimental effects on seminal parameters in infertile men, thus highlighting the need for cessation programs as part of fertility treatment protocols. Encouraging smoking cessation could substantially improve semen quality and fertility outcomes in this population.

## 1 Introduction

Infertility is characterized as the inability of couples within the reproductive age spectrum to conceive despite engaging in regular, unprotected coitus for a span exceeding one year. A thorough examination of 25 population-based studies demonstrated that approximately 9% of individuals of reproductive age worldwide, across both advanced and developing nations, are impacted by infertility. Significantly, male factors play a role in half of these instances (1). Infertility exerts a substantial global influence, whereby it affects an estimated 70 million individuals throughout the world. The World Health Organization (WHO) has recognized infertility as a paramount public health challenge, thus highlighting the need for enhanced clinical focus. Research suggests that a worldwide decrease in sperm quality, including decreased sperm counts among men of reproductive age, is linked to environmental pollutants, occupational stress, and suboptimal dietary habits (2). This Introduction section emphasizes the importance of exploring infertility, acknowledges the current insights into its prevalence and causative factors, and positions this study as a significant step forward in deepening our understanding of the factors contributing to infertility and possible interventions. By refining the description of infertility to avoid repetitiveness, the narrative can become more clear, thus focusing on the core criteria for defining infertility.

Routine semen analysis is essential before assisted reproductive medicine treatment. Evidence-based recommendations highlight the importance of a comprehensive medical history, detailed physical examination, and assessment of factors affecting semen quality as initial evaluative measures for male patients. Abnormalities in semen analysis can provide valuable insights into various aspects of male infertility, which often results from a complex interplay of genetic and socioenvironmental factors. A significant body of research, including a 2021 meta-analysis on molecular genetics, has identified genetic underpinnings for approximately half of all infertility cases in men, thus manifesting in reduced sperm count, sperm viability, or increased morphologically abnormal sperm (3). Despite these advancements, the specific mechanisms of male genetic infertility require further exploration (4). In addition to genetic factors, lifestyle factors significantly impact male fertility. In particular, smoking has been extensively shown to detrimentally affect male reproductive health, with smokers often requiring more *in vitro* fertilization (IVF) attempts to achieve conception (5). Lead and cadmium, which are prevalent in tobacco smoke, contribute to decreased male fertility through oxidative stress pathways, thus damaging sperm DNA and reducing sperm production (6). This finding has been corroborated by population-based epidemiological studies and animal experiments (7). Therefore, lifestyle modifications, such as maintaining a balanced diet, weight control, moderate exercise, and minimizing exposure to environmental pollutants, are advocated to enhance men’s sexual health and reproductive outcomes (8).

Male infertility is typically categorized into primary infertility, wherein individuals have been infertile both previously and currently, and secondary infertility, wherein individuals were fertile in the past but are currently experiencing infertility issues (1, 9). Although earlier research, including studies by Al-Turki in 2014 and 2016 (1), have targeted specific demographics, such as infertile men in Saudi Arabia, these investigations were limited by small sample sizes (258 and 425 cases, respectively) and data that may no longer reflect current trends (spanning the years of 2008–2013). This scenario suggests that the information collected during this time period may not accurately represent the latest developments or changes in infertility patterns, thus necessitating more recent and broader studies to understand the current state of male infertility. Therefore, we conducted the present study to offer valuable and superior research data in this field. Reports indicate that the causes of secondary infertility in men may include surgical procedures, radiotherapy, varicocele and aging, which are also significant factors in the development of secondary infertility (10). Given the prevalence of smoking as a lifestyle habit among adult men throughout the world, with more than one-third of them using tobacco (Tobacco-WHO), an understanding of its impact on male infertility has become particularly pertinent.

## 2 Methods

### 2.1 Patients

**Figure 1:**
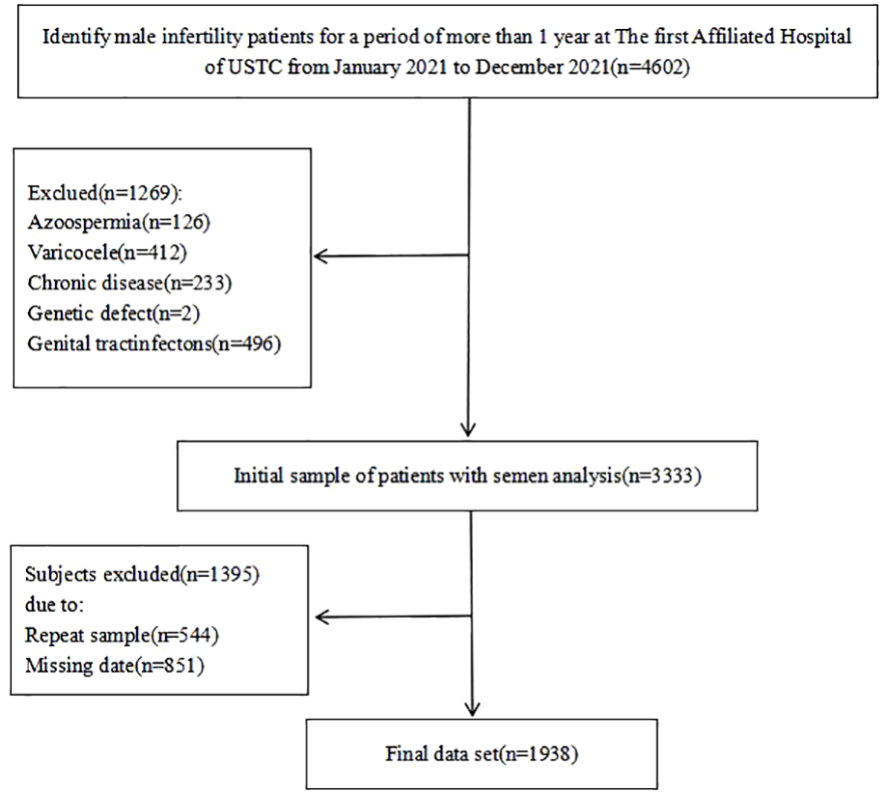
Flow diagram for the selection of the eligible study population.

To assess the link between cigarette smoking and semen quality among infertile men, a cross-sectional analysis was performed from January to December 2021 in Anhui Province, China (refer to **Figure 1** for details). The eligibility criteria required male participants to be diagnosed with infertility, with an absence of concurrent reproductive abnormalities (e.g., varicocele, syringomyelia, cryptorchidism, and inguinal hernia) or hormonal abnormalities. The female partners were expected to exhibit regular fertility cycles indicative of ovulatory function and normal uterine structures. Initially, the study aimed to include a cohort of 4,602 men. However, during data verification, 1,269 participants were excluded for various reasons, including azoospermia (n=126), varicocele (n=412), reproductive tract infections (n=496), chronic severe debilitating conditions (n=233), or specific genetic abnormalities (n=2). Notably, some participants were disqualified for multiple reasons (n=544), and a significant number (n=851) were excluded for not providing complete information on their somking consumption habits. Ultimately, the study included 1,938 men, all of whom presented with abnormal semen parameters in conjunction with the 5th edition of the WHO criteria Ethical approval for this research was obtained from the Ethics Committee of the First Affiliated Hospital of USTC, China (Ethics Approval Number: 2021-RE-072).

The study delineated two primary groups based on smoking behavior: smokers (who were further divided into moderate smokers, consuming 1-10 cigarettes per day (11), and heavy smokers, consuming more than 10 cigarettes per day (12)) and nonsmokers (nonsmokers included quitters and never-smokers). This classification was pivotal in examining the potential gradations in semen quality across different levels of tobacco exposure among the infertile male population.

### 2.2 Semen parameters

Prior to the collection of semen samples, participants were instructed to urinate to ensure a clean urethra and advised to abstain from ejaculation for a period of two to seven days. To further ensure sample purity, an increased intake of water was recommended on the day preceding the collection. The WHO Laboratory provided sterile plastic containers for the collection of semen samples. Semen volume was measured by weight (milliliters), and samples were allowed to liquefy at 37°C for 30 minutes before analysis, thus adhering to the WHO 2010 standards. The analysis included evaluations of sperm volume, concentration, progressive motility, and nonprogressive motility. Cytological staining was conducted by using the Diff-Quick staining kit supplied by Anke Biotechnology Co., Ltd., thus facilitating the assessment of sperm morphology through microscopic examination of more than 200 spermatozoa. The identification of anti-sperm antibodies (AsA) employed the mixed antiglobulin reaction (MAR) method, Which was also provided by Anke Biotechnology (Hefei, China). Leukocytospermia was determined when leukocyte counts exceeded 1 × 10^6^ ml^-1^.

### 2.3 Clinical characteristics

Body mass index (BMI), which is a universally recognized metric for assessing obesity levels, was calculated for each participant by using the formula BMI = weight (kg)/height^2^ (m^2^), based on the WHO China BMI thresholds from the 2004 WHO Expert Consultation.

### 2.4 Research design

Participants completed a detailed questionnaire regarding their smoking habits to facilitate data collection. Smoking frequency was recorded as the number of cigarettes smoked per day, dividing participants into quitters (nonsmokers), moderate smokers (1-10 cigarettes per day), and heavy smokers (more than 10 cigarettes per day). The classification also considered the participants’ reproductive history, distinguishing those with primary infertility (those who were unable to conceive for more than a year without contraception) from those with secondary infertility (those who previously had children but were unable to conceive). Additional clinical characteristics, such as age, BMI, ethnicity, education level, duration of infertility, alcohol consumption, late-night snacking habits, dietary patterns, working hours, and sleep duration, were also collected. Semen analysis followed the protocols outlined in the 2010 WHO manual, with abnormal semen parameters defined as a semen volume less than 1.5 ml, a sperm concentration less than 15×10^6^ ml, and a motility pattern less than 4%.

### 2.5 Statistical analysis

The statistical analysis was conducted by using Prism 9.0 software (San Diego, CA), with a significance threshold set at P<0.05. Quantitative variables are presented as the mean ± standard deviation (SD) for normally distributed data, whereas nonnormally distributed data are presented as the median and interquartile range (min–max or ideally with 25th-75th percentiles). The Kolmogorov-Smirnov test was employed to determine the distribution of the variables. For normally distributed data, one-way ANOVA or t tests were used, whereas Kruskal-Wallis or Mann-Whitney U tests were used for nonnormally distributed data. Categorical variables were analyzed by using the chi-squared test, with Fisher’s exact test invoked under conditions necessitating its use. This comprehensive statistical framework ensured the meticulous examination of the relationship between smoking habits and semen characteristics, thus facilitating a nuanced understanding of their interplay within the context of male infertility.

## 3 Results

**Table 1** presents a detailed breakdown of the characteristics distinguishing men with primary infertility from men with secondary infertility among a study cohort of 1,938 participants. Men with primary or secondary infertility had an average age of 30.4 ± 4.6 years, with a notable age difference observed between the groups; specifically, individuals in the secondary infertility group were older (averaging 32.4 ± 5.3 years) compared to 29.7 ± 4.1 years in the primary infertility group. This age disparity was significant, with 10.6% of the patients in the secondary infertility group being over 40-years-old versus only 2.5% in the primary group. Lifestyle factors, including diet, alcohol consumption, duration of infertility, work hours, and sleep, showed no significant differences between the two groups in terms of their influence on infertility type. However, education level markedly differed; specifically, higher education was more prevalent in the primary infertility group. Interestingly, primary infertile men reported of a greater frequency of night snacking than their secondary counterparts. From a clinical perspective, primary infertile men also displayed superior sperm progressive motility and a lower incidence of smoking, yet they exhibited lower sperm concentrations, thus highlighting intricate relationships between lifestyle choices, clinical characteristics, and infertility type.

**Table 1:** Descriptive and characteristic statistics.

Clinical characteristics	Total (n=1938)	Primary infertile men (n=1430)	Secondary infertile men(n=508)	P
Age (year),mean ± s.d.	30.4 ± 4.6	29.7 ± 4.1	32.4 ± 5.3	<0.001
<40, n (%)	95.3 (1848)	97.5 (1394)	89.4 (454)	<0.001
≥40, n (%)	4.7 (90)	2.5 (36)	10.6 (54)	
BMI (kg/m^2), mean ± s.d.	24.9 ± 4.0	25.0 ± 4.0	24.9 ± 4.1	0.52
<24, n (%)	42.4 (822)	42.3 (605)	42.7 (217)	0.87
≥24, n (%)	57.6 (1116)	57.7 (825)	57.3 (291)	
Nation,n (%)				0.13
Han	99.6 (1930)	99.7 (1426)	99.2 (504)	
Other	0.4 (8)	0.3 (4)	0.8 (4)	
Education,n (%)				<0.0001
Primary school	1.5 (29)	1.2 (17)	2.4 (12)	
Junior high school	20.9 (404)	18.7 (267)	27.0 (137)	
High school	18.7 (363)	18.3 (262)	19.9 (101)	
College/University	58.9 (1142)	61.8 (884)	50.8 (258)	
Duration of infertility,n (%)				0.08
≤1 year	74.8 (1450)	75.7 (1082)	72.4 (368)	
>1 year, <3 year	13.4 (259)	13.5 (193)	13.0 (66)	
≥3 year	11.8 (229)	10.8 (155)	14.6 (74)	
Alcohol status,n (%)				0.16
Drinkers	52.2 (1012)	51.3 (733)	54.9 (279)	
Non-drinkers	47.8 (926)	48.7 (697)	45.1 (229)	
Smoking status,n (%)				0.006
non-smokers	55.1 (1067)	57.1 (817)	49.2 (250)	
Moderate smoker	29.3 (568)	28.3 (405)	32.1 (163)	
Heavy smoker	15.6 (303)	14.6 (208)	18.7 (95)	
Frequency of night snack intake,n (%)				0.009
0/week	31.7 (614)	29.4 (420)	38.2 (194)	
1-3/week	64.7 (1254)	66.8 (956)	58.7 (298)	
>3/week	3.6 (70)	3.8 (54)	3.1 (16)	
Dietary habits,n (%)				0.27
Regular diet (three meals per day)	65.7 (1274)	65.0 (930)	67.7 (344)	
Irregular diet	34.3 (664)	35.0 (500)	32.3 (164)	
Work time,n (%)				0.21
<8 (h/d)	34.4 (667)	33.2 (475)	37.8 (192)	
8-10 (h/d)	47.7 (924)	49.3 (705)	43.1 (219)	
>10 (h/d)	17.9 (347)	17.5 (250)	19.1 (97)	
Sleep time,n (%)				0.12
≤8 (h/d)	73.7 (1428)	74.6 (1067)	71.1 (361)	
>8 (h/d)	26.3 (510)	25.4 (363)	28.9 (147)	
Semen parameters				P
Semen volume (ml),median (Q1,Q3)	3.1 (2.2,4.2)	3.1 (2.2,4.3)	3.0 (2.1,4.1)	0.17
Semen volume<1.5 (ml),n (%)	8.6 (166)	8.5 (122)	8.7 (44)	0.93
Sperm concentration (×10^6/ml),median (Q1,Q3)	67.7 (35.6,118.4)	65.3 (34.1,116.2)	72.4 (42.6,125.2)	0.0093
Sperm concentration<15×10^6/ml,n (%)	9.2 (179)	9.9 (141)	7.5 (38)	0.11
Progressive motility (%),median (Q1,Q3)	45.4 (33.1,57.7)	43.4 (30.0,55.1)	42.8 (28.7,54.1)	<0.0001
Progressive motility<32%,n (%)	28.1 (545)	29.9 (428)	23.0 (117)	0.003
Normal morphology (%),median (Q1,Q3)	6 (5,8)	7 (5,8)	6 (5,8)	0.33
Normal morphology<4%,n (%)	9.8 (179)	10.7 (143)	7.5 (36)	0.05
Asthenoteratozoospermia,n (%),n (%)	54.6 (1058)	55.5 (794)	52.0 (264)	0.17
Oligoasthenoteratozoospermia,n (%)	4.6 (90)	5.2 (75)	3.0 (15)	0.035
Leukocytic count (×106/ml),median (Q1,Q3)	0.1 (0.0,0.5) (n=998)	0.1 (0.0,0.5) (n=755)	0.1 (0.0,0.5) (n=243)	0.91
AsA (%),median (Q1,Q3)	2.0 (1.0,4.0) (n=821)	2.0 (1.0,4.0) (n=597)	2.0 (1.0,4.0) (n=224)	0.32

BMI, was calculated using P values that came from the chi-square test. If the data are regularly distributed, they are shown as the mean ± standard deviation, and for categorical variables, as n (%), and for continuous variables, as medians (Q1, Q3). 25th percentile is Q1. Q3 is the 75th percentile.

**Table 2** shows the associations between various factors and primary versus secondary infertility. Significantly, the data demonstrated that heavy smokers were notably older than light smokers, with individuals who had quit smoking also displaying a tendency toward older age. A marked statistical correlation was identified among men experiencing infertility with respect to the frequency of nighttime snacking and consistency in dietary habits. Intriguingly, as smoking intensity increased, there was a noticeable increase in the incidence of irregular dietary patterns and late-night snacking, particularly among those with primary infertility, who reported of a greater prevalence of these behaviors than individuals with secondary infertility. Furthermore, an analysis of semen parameters demonstrated that a progressive reduction in semen volume was correlated with increased smoking frequency, which was a trend observed in both primary and secondary infertility cohorts. This pattern highlights the broader implications of lifestyle factors for reproductive health and indicates the potential for mitigating infertility risks through targeted lifestyle modifications. More specifically, there was a substantial relationship between smoking status and sperm concentration in primary infertile men (nonsmokers, moderate smokers, and heavy smokers), with sperm concentration decreasing progressively as smoking intensity increased. Conversely, in the case of secondary infertility, a significant correlation was identified between sperm forward motility and cigarette smoking, thus demonstrating a progressive reduction in sperm motility with increasing cigarette consumption.

**Table 2:** Descriptive statistics based on the smoking status.

	Primary infertile men(N=1430)				Secondary infertile men(N=508)			
	non-smokers (n=817)	Moderate smokers(n=405)	Heavy smokers(n=208)	P	non-smokers(n=250)	Moderate smokers(n=163)	Heavy smokers(n=95)	P
Age(year),mean ± s.d.	29.8 ± 3.9	29.2 ± 4.0	30.1 ± 4.8	0.04	32.4 ± 5.3	31.5 ± 5.3	33.7 ± 5.2	0.001
<40,n(%)	97.4(795)	98.3(398)	96.1(200)	0.28	89.6(224)	92.0(150)	84.2(80)	0.14
≥40,n(%)	1.5(21)	1.7(7)	3.8(8)		10.4(26)	8.0(13)	15.8(15)	
BMI(kg/m^2),mean ± s.d.	24.8 ± 3.7	25.3 ± 4.4	25.1 ± 4.4	0.24	24.6 ± 3.5	25.0 ± 4.4	25.7 ± 5.0	0.39
<24,n(%)	43.0(351)	49.4(165)	42.8(89)	0.74	42.8(107)	44.8(73)	36.8(35)	0.45
≥24,n(%)	57.0(465)	50.6(240)	57.2(119)		57.2(143)	55.2(90)	63.2(60)	
Duration of infertility,n(%)				0.11				0.18
≤1 year	76.2(622)	76.8(311)	71.6(149)		72.4(181)	72.4(118)	72.6(69)	
>1 year,<3 year	14.4(118)	11.9(48)	13.0(27)		13.2(33)	16.0(26)	7.4(7)	
≥3 year	9.4(77)	11.3(46)	15.4(32)		14.4(36)	11.6(19)	20.0(19)	
Frequency of night snack intake,n(%)				0.0001				<0.0001
0/week	33.5(274)	25.2(102)	21.2(44)		50.0(125)	28.2(46)	24.2(23)	
1-3/week	63.8(521)	70.4(285)	72.1(150)		47.6(119)	68.7(112)	70.5(67)	
>3/week	2.7(22)	4.4(18)	6.7(14)		2.4(6)	3.1(5)	5.3(5)	
Dietary habits,n(%)				<0.0001				<0.0001
Regular diet(three meals per day)	72.3(591)	59.5(241)	47.1(98)		80.4(201)	60.7(99)	46.3(44)	
Irregular diet	27.7(226)	40.5(164)	52.9(110)		19.6(49)	39.3(64)	53.7(51)	
Work time, n(%)				0.06				0.89
<8(h/d)	32.3(264)	34.3(139)	34.6(72)		38.0(95)	38.0(62)	36.8(35)	
8-10(h/d)	52.3(427)	46.1(187)	43.8(91)		44.0(110)	40.5(66)	45.3(43)	
>10(h/d)	15.4(126)	19.5(79)	21.6(45)		18.0(45)	21.5(35)	17.9(17)	
Sleep time, n(%)				0.36				0.06
≤8(h/d)	76.0(621)	72.3(293)	73.6(153)		75.6(189)	65.0(106)	69.5(66)	
>8(h/d)	24.0(196)	27.7(112)	26.4(55)		24.4(61)	35.0(57)	30.5(29)	
Semen parameters								
Semen volume(ml),median(Q1,Q3)	3.3(2.3,4.4)	3.0(2.2,4.1)	2.8(2.0,4.0)	0.01	3.2(2.2,4.5)	3.0(2.1,4.1)	2.8(1.8,3.7)	0.008
Semen volume<1.5(ml),n(%)	7.6(62)	8.6(35)	12.0(25)	0.67	6.8(17)	9.2(15)	12.6(12)	0.22
Sperm concentration(×10^6/ml), median(Q1,Q3)	68.6(36.0,121.7)	60.7(31.4, 101.8)	59.2(28.8,116.2)	0.01	77.9(45.6,129.4)	69.2(34.2, 120.8)	72.3(43.2,127.8)	0.18
Sperm concentration<15×10^6/ml, n(%)	7.7(63)	12.1(49)	13.9(29)	0.005	5.6(14)	8.6(14)	10.5(10)	0.24
Progressive motility(%),median (Q1,Q3)	41.5(28.8,52.8)	43.4(28.5,55.0)	45.5(28.9,55.7)	0.25	48.1(36.3,58.5)	46.4(34.2,60.1)	44.7(29.3,56.3)	0.01
Normal morphology(%),median (Q1,Q3)	7(4,8)	6(5,8)	7(5,8)	0.7	6(5,8)	6(5,8)	6(4,8)	0.84
Normal morphology<4%,n(%)	23.7(193)	23.7(96)	19.7(41)	0.46	23.2(58)	16.6(27)	27.4(26)	0.10

P values are derived, unless otherwise noted, using analysis of variance.

**Table 3** presents the significant correlations observed between sperm concentration and various semen parameters. In the primary infertility group, a substantial correlation was identified between smoking and sperm concentration, even after adjusting for factors such as age, BMI, abstinence duration, alcohol consumption, infertility duration, intake of night-time snacks, dietary habits, and working hours. However, no significant associations were detected between smoking and other semen characteristics, including volume, spermatozoa count, and morphology. Within the group experiencing secondary infertility, smoking status was not significantly related to semen parameters such as semen volume or total sperm count. Nevertheless, a noteworthy correlation emerged between the extent of smoking and the rate of progressive sperm motility.

**Table 3:** Probability ratios with 95% confidence intervals for aberrant semen scope at varying degrees of tobacco use.

	Primary infertile men			Secondary infertile men		
	non-smokers (n=817)	Moderate smokers(n=405)	Heavy smokers(n=208)	non-smokers (n=250)	Moderate smokers(n=163)	Heavy smokers(n=95)
Semen volume						
Crude	ref	1.15(0.74,1.76)	1.66(1.00,2.69)	ref	1.39(0.67, 2.87)	1.98(0.89, 4.30)
P		0.52	0.04		0.37	0.09
Adjusted	ref	1.13(0.71,1.76)	1.53(0.90,2.54)	ref	1.16(0.53, 2.52)	1.18(0.48, 2.77)
P		0.61	0.11		0.71	0.71
Sperm concentration						
Crude	ref	1.65(1.11,2.44)	1.94(1.20,3.07)	ref	1.58(0.73, 3.44)	1.98(0.83,4.60)
P		0.01	0.005		0.24	0.11
Adjusted	ref	1.60(1.05, 2.40)	1.88(1.14,3.05)	ref	1.38(0.61, 3.11)	1.65(0.65,4.06)
P		0.03	0.01		0.43	0.28
Total count						
Crude	ref	1.39(0.94, 2.04)	1.74(1.09,2.73)	ref	1.48(0.71,3.10)	1.92(0.836,4.26)
P		0.09	0.02		0.29	0.11
Adjusted	ref	1.39(0.92, 2.07)	1.76(1.08,2.81)	ref	1.27(0.58,2.75)	1.67(0.68,3.93)
P		0.11	0.02		0.55	0.25
Progressive motility						
Crude	ref	0.98(0.75, 1.27)	0.91(0.65,1.276)	ref	0.51(0.31,0.83)	0.54(0.29,0.96)
P		0.86	0.58		0.007	0.04
Adjusted	ref	1.10(0.845,1.44)	1.00(0.70,1.42)	ref	0.53(0.31,0.87)	0.53(0.28,0.99)
P		0.5	0.99		0.01	0.05
Total motility						
Crude	ref	0.88(0.69,1.13)	0.88(0.64,1.21)	ref	0.61(0.39,0.96)	0.79(0.46,1.3)
P		0.32	0.45		0.03	0.37
Adjusted	ref	0.95(0.73,1.23)	0.93(0.66,1.30)	ref	0.63(0.39,1.01)	0.79(0.44,1.37)
P		0.7	0.67		0.06	0.40
Nomal morphology						
Crude	ref	0.88(0.58,1.32)	1.26(0.77,2.01)	ref	0.41(0.15,0.98)	0.94(0.38,2.12)
P		0.55	0.34		0.06	0.89
Adjusted	ref	0.96(0.62,1.46)	1.46(0.87,2.38)	ref	0.37(0.13,0.93)	0.88(0.33,2.16)
P		0.86	0.14		0.05	0.79

Men having the following WHO-recognized semen quality values were used as the reference group for parameter analysis: semen volume ≥ 1.5 ml, sperm concentration ≥ 15 × 10^6^/ml, and progressive motility sperm ≥ 32 × 10^6^/ml. Crude: unmodified model Forage, BMI, period of abstinence, alcohol intake, length of infertility, how many nocturnal snacks consumed, dietary practices, and working hours were all changed in the model; ref stands for the reference.

## 4 Discussion

In this comprehensive cross-sectional study involving 1,938 infertile men from China, we investigated the association between smoking habits and semen quality by stratifying participants into nonsmokers, moderate smokers (1-10 cigarettes per day), and heavy smokers (more than 10 cigarettes per day). Our findings highlight the significant impact of smoking on semen parameters and demonstrates a marked correlation between tobacco use and diminished semen quality among both men with primary infertility and men with secondary infertility (13). Specifically, data analysis demonstrated that nonsmokers exhibited higher sperm concentrations than smokers, with a notable decrease in sperm concentration as smoking intensity increased. For instance, the median sperm concentration was 67.7 (35.6, 118.4) for the total cohort, with a discernible decrease among heavy smokers. Moreover, the study illuminated the differential effects of smoking on primary versus secondary infertility. In primary infertility cases, a significant association was found between smoking and semen concentration, wherein heavy smokers demonstrated the lowest sperm concentration and viability (3). Conversely, secondary infertility was notably linked to reduced sperm forward motility, thus showcasing a progressive decline with increased smoking levels (14). This dichotomy suggests that smoking has multifaceted detrimental effects on male reproductive health, depending on the infertility type. Our study also investigated the broader implications of lifestyle factors on infertility. Notably, heavy smokers were significantly older than light smokers, and the incidence of secondary infertility was greater among older participants, thus underscoring the compounded effect of age and long-term smoking on fertility outcomes. Furthermore, lifestyle patterns, including alcohol consumption, late-night snacking, and dietary habits, exhibited a significant correlation with smoking intensity, particularly accentuated in primary infertility cases (15). Interestingly, the primary infertility group, characterized by higher educational levels, showed more frequent late-night snacking habits, hinting at a potential link between socio-economic status, lifestyle patterns, and infertility. These patterns suggest that the cumulative impact of lifestyle factors, coupled with aging, might predispose individuals to secondary infertility, underscoring the need for a nuanced understanding of how lifestyle choices impact reproductive health over time.

This study significantly advances the discourse on the impact of smoking on male fertility, thus providing empirical evidence that underscores the detrimental effects of cigarette consumption on seminal quality. This scenario highlights the imperative for men facing infertility challenges to reconsider lifestyle choices (particularly smoking habits) as part of their fertility treatment plan (16). Our research highlights the importance of a proactive stance in fertility clinics, wherein health care professionals should counsel patients on the benefits of quitting smoking to improve seminal quality and, consequently, fertility outcomes.

By delving into the nuanced relationship between lifestyle factors and male infertility, this investigation enriches the scholarly dialog and sets the stage for further research aimed at elucidating the complex dynamics that are occurring. The marked correlation between smoking and diminished semen parameters (specifically, sperm concentration in cases of primary infertility and sperm motility in cases of secondary infertility) emphasizes the critical need for targeted interventions and public health campaigns to counteract the harmful effects of smoking on reproductive health (17).

This study is pioneering in its thorough examination of smoking patterns among Chinese men suffering from primary and secondary infertility, whereby it categorized participants into smokers and nonsmokers and further distinguished smokers by intensity of consumption (18). Similar to the findings from Caster D et al.’s study, which reported significant reductions in sperm concentration among smokers compared to nonsmokers within a sample of 648 infertile males, our analysis extends these observations. We found that smoking adversely affects sperm viability and morphology to a lesser extent but significantly reduces sperm concentration, with a clear dose-response relationship observed between smoking intensity and sperm quality degradation (19). A particularly alarming result is the discovery that heavy smokers within the primary infertility cohort exhibited the lowest sperm concentration and viability, thus illustrating a direct correlation between smoking severity and a decline in sperm health.

Additionally, our findings demonstrated that individuals with secondary infertility were generally older and had a longer history of smoking, thus suggesting a cumulative effect of tobacco exposure over time. This prolonged engagement with smoking, coupled with age-related factors, contributes to an increased incidence of secondary infertility, thus indicating a compounded risk associated with long-term smoking habits (4). In summary, this investigation not only contributes valuable insights into the effects of smoking on male fertility but also advocates for a holistic approach to fertility treatment, thus emphasizing lifestyle modifications as a crucial component of improving reproductive health outcomes.

In this study, we meticulously examined the interplay between several lifestyle and health factors, including age, body mass index (BMI), alcohol consumption, duration of infertility, dietary patterns, and work and sleep duration, and their impact on semen quality (20). Through our analysis, we established pronounced correlations, notably with age, dietary habits, and smoking behaviors, which demonstrated their crucial influence on male fertility.

Our findings highlight the significant role of age in semen quality. With societal trends shifting toward delayed marriages, age has surfaced as being a paramount determinant affecting male reproductive health. The testes, which are pivotal for spermatogenesis (the formation of mature spermatozoa) and steroidogenesis (the production of testosterone, which is vital for male sexual functions) are impacted by the aging process. This study provides more information on how aging correlates with a progressive decrease in testosterone levels, which is indicative of a reduction in stromal cell activity responsible for testosterone synthesis (7). An age-related decrease in semen quality was evident, with individuals older than 40 years showing increased instances of abnormal semen volume, motility, viability, and sperm kinematics. This finding aligns with Agarwal et al.’s observations of diminished sperm concentration, motility, and volume as age advances, thus highlighting the undeniable impact of aging on fertility (15).

Furthermore, our data demonstrated a notable difference in age between men with primary versus secondary infertility, thus illustrating a demographic trend that supports the literature suggesting that secondary infertility is more prevalent among older men. Specifically, our analysis revealed that the mean ages of ex-smokers were 29.8 ± 3.9 years for men with primary infertility and 32.4 ± 5.3 years for men with secondary infertility. Similarly, the average age of heavy smokers in the primary infertility group was 30.1 ± 4.8 years, whereas that of their counterparts in the secondary infertility group was 33.7 ± 5.2 years. These findings highlight the potential cumulative effect of smoking over time, which is compounded by age-related factors, on fertility outcomes. It is important to clarify that our study did not conduct detailed age subgroup analyses to further explore these observed trends. The decision not to perform such analyses was based on the scope and objectives of our initial research design, which aimed to broadly assess the relationship between smoking habits and infertility without delving into the specific impacts within narrower age ranges. This approach, while insightful, suggests that further research is necessary to dissect the nuances of how age influences the relationship between smoking and fertility across different stages of male infertility. In contrast to the findings of Rehman R et al. (21), who reported no significant differences in age, BMI, or body fat between smokers and nonsmokers within the infertile cohort, our study demonstrated a distinct age trend among heavy smokers, thus indicating that those suffering from secondary infertility tended to be older. However, similar to their findings, we observed no significant correlation between BMI and male infertility in our cohort. This lack of significant BMI correlation, coupled with our insights into age differences, highlights the complex interplay of factors influencing male fertility and also highlights the need for a multifaceted approach in future research.

Our comprehensive analysis delved into the intricate relationship between various lifestyle and health parameters-such as age, Body Mass Index (BMI), alcohol consumption, duration of infertility, and specific dietary habits-and their impact on semen quality. While our investigation identified significant correlations with age, dietary practices, and smoking, offering new insights into the multifactorial influences on male fertility, it is important to note that our study did not directly compare BMI between infertile patients and a control group of normal, fertile men. The decision not to compare BMI across these groups was driven by our focused research objectives, which aimed to understand the dynamics within an infertile cohort rather than contrast these with the general population. Although BMI showed no significant correlation with male infertility within our specific study population, this finding does not preclude the importance of BMI as a factor in male reproductive health more broadly. Previous studies have suggested varied impacts of BMI on semen quality, but our analysis contributes to the ongoing discourse by emphasizing that within our cohort of infertile men, BMI was not a distinguishing factor in fertility outcomes. By integrating these specific data points, our analysis not only deepens the understanding of lifestyle factors on male fertility but also underscores the importance of considering a range of factors-including age, diet, and smoking cessation—in improving reproductive outcomes (22). This study advocates for tailored interventions and comprehensive fertility evaluations that consider these critical factors, offering a pathway toward optimizing male reproductive health.

In contrast to previous studies that suggested that BMI has a negligible effect on semen quality, our research, which included an analysis of 206 men meeting our stringent criteria, did not establish a significant correlation between increased BMI and semen parameters. However, our study demonstrated pronounced associations between dietary patterns and semen quality. Cross-sectional research involving healthy Taiwanese men over 18 years of age highlighted the detrimental effects of high-sugar and high-carbohydrate diets on sperm motility and concentration, respectively (P = 0.012 and P = 0.025 for total sperm motility and progressive motility, respectively; P = 0.001 for sperm concentration). Furthermore, our findings suggest a negative impact of high sodium and saturated fat intake on sperm motility and overall sperm health (23), thus reinforcing the importance of nutritional choices in male fertility.

Particularly noteworthy is the positive correlation between sperm concentration and a healthy dietary pattern, as well as the frequency of sexual activity among men with suboptimal semen quality. This underscores the pivotal role of diet in reproductive health, which corresponds to Craig Niederberger’s emphasis on the benefits of a nutritious diet for enhancing semen quality (24), especially in men with compromised semen parameters.

Despite the robustness of our findings, it is important to acknowledge the limitations inherent in our study’s design. The regional specificity of our participant pool may restrict the generalizability of our conclusions. Additionally, the cross-sectional nature of our survey could introduce recall bias, and the reliance on self-reported data for smoking habits may not capture the full spectrum of tobacco exposure. The potential for residual confounding factors remains a challenge, as is common in observational studies, thus possibly affecting the observed associations.

However, the strengths of our study, including its prospective design, the clear differentiation between primary and secondary infertility, and the comprehensive data collection through detailed questionnaires—allow for a nuanced understanding of the interplay between lifestyle factors and infertility (21). By considering both smoking and alcohol consumption and their cross-correlation, we were able to isolate their independent effects on fertility outcomes.

Overall, our research contributes significantly to the ongoing discourse on male reproductive health by demonstrating the adverse effects of smoking on semen quality among Chinese men with infertility. By demonstrating the clear link between smoking and diminished semen concentration in primary infertile men, as well as decreased sperm viability in secondary infertile cases, our study highlights critical areas for intervention and further research. These findings advocate for a holistic approach to fertility treatment, thus emphasizing the need for lifestyle modifications alongside medical interventions to optimize reproductive outcomes.

## Data availability statement

The raw data supporting the conclusions of this article will be made available by the authors, without undue reservation.

## Ethics statement

This study was approved by The First Affiliated Hospital of the USTC Ethical Committee (2021-RE-072). Written informed consent was obtained from all participants. The studies were conducted in accordance with the local legislation and institutional requirements. The participants provided their written informed consent to participate in this study. Written informed consent was obtained from the individual(s) for the publication of any potentially identifiable images or data included in this article.

## Author contributions

SF: Data curation, Software, Writing – original draft, Conceptualization, Methodology, Project administration, Resources. ZZ: Data curation, Formal analysis, Investigation, Methodology, Software, Writing – original draft. HW: Data curation, Investigation, Methodology, Software, Writing – original draft. LL: Writing – review & editing. BX: Conceptualization, Project administration, Writing – review & editing.
